# Lenalidomide in Combination with Dexamethasone in Elderly Patients with Advanced, Relapsed or Refractory Multiple Myeloma and Renal Failure

**DOI:** 10.4084/MJHID.2013.037

**Published:** 2013-06-03

**Authors:** Patrizia Tosi, Barbara Gamberi, Barbara Castagnari, Anna Lia Molinari, Paolo Savini, Michela Ceccolini, Monica Tani, Anna Merli, Manuela Imola, Anna Maria Mianulli, Claudia Cellini, Simona Tomassetti, Francesco Merli, Pierpaolo Fattori, Alfonso Zaccaria

**Affiliations:** 1 Hematology Unit, Infermi Hospital, Rimini Italy; 2 Hematology Unit, Department of Oncology and Advanced Thechnologies, S Maria Nuova Hospital IRCSS Reggio Emilia Italy; 3 Hematology Unit, S. Maria Delle Croci Hospital, Ravenna Italy; 4 Internal Medicine Unit, Faenza Hospital Italy; 5 IRCCS IRST Meldola Italy

## Abstract

Salvage therapy of elderly patients with advanced, relapsed and refractory multiple myeloma (MM) is often limited by poor marrow reserve and multi-organ impairment. In particular, renal failure occurs in up to 50% of such patients, and this can potentially limit the therapeutic options. Both thalidomide and bortezomib have proven effective in these patients, with an acceptable toxicity, while, in clinical practice, lenalidomide is generally not considered a first-choice drug for MM patients with renal failure as early reports showed an increased hematological toxicity unless appropriate dose reduction is applied. Aim of this study was a retrospective evaluation of the efficacy of the combination Lenalidomide + Dexamethasone in a population of elderly MM patients treated in 5 Italian Centers. The study included 20 consecutive MM patients (9 M, 11 F, median age 76.5 years) with relapsed (N= 6) or refractory (N=13) MM and moderate to severe renal failure, defined as creatinine clearance (Cr Cl) < 50ml/min. Four patients were undergoing hemodyalisis at study entry. 85 % of the patients had been previously treated with bortezomib-containing regimens. Lenalidomide dose was adjusted according to renal function and patients clinical conditions Median treatment duration was 16 months (1–22), therapy was interrupted after 1 21-day cycle in 2 patients. Grade III–IV neutropenia was observed in 7 patients (35%); grade III–IV non hematological toxicity was recorded in 3 cases (28%). A > partial response was observed in 8 patients (40%), 1 of whom obtained a VGPR; 4 additional patients achieved a minor response. Median response duration was 16 months (range 2–19+ months). A complete and partial renal response was obtained in 4 and 3 patients, respectively, all of them were responsive to Lenalidomide-dexamethasone According to our data, LEN+DEX has shown efficacy and acceptable toxicity in this population of elderly patients with advanced MM and renal failure

## Introduction

Multiple myeloma (MM) is a clonal B cell malignancy characterized by a progressive clinical course, usually within 3 to 5 years from diagnosis.^1^ The introduction of novel agents such as thalidomide, bortezomib or lenalidomide in the framework of high-dose or conventional chemotherapy programs has significantly improved patients’ outcome and survival.^2^ Patients who are refractory or those who relapse after new agents-containing regimens, however, do still represent a therapeutic challenge,^3^ especially in case of advanced age and presence of co-morbidities. Renal failure occurs in approximately 20–30% of MM patients at diagnosis and in more than 50% of patients with advanced disease.^4^ Salvage therapy in these patients is difficult, due to the necessity to avoid potentially nephrotoxic drugs or compounds that are excreted by the kidney and, until a few years ago, the options were limited to VAD or other high-dose dexamethasone containing regimens. Recently, however, both thalidomide^5^ and bortezomib^6^ have proven effective in reverting renal failure in MM patients responding to treatment, with an acceptable toxicity profile. Lenalidomide is frequently considered a second-choice drug for MM patients with renal failure as it is catabolized through the kidney^7^ and its use has been initially associated with an increased hematological toxicity in patients with impaired renal function.^8^–^11^ Recent observations, however, have pointed out that improvement of renal function can be achieved, in case a response is obtained,^10^–^13^ and that side effects are easily manageable in case a proper dose reduction is performed.^10^–^15^ Aim of the present study was to investigate the efficacy and the toxicity profile of lenalidomide-dexamethasone combination in a series of elderly patients with advanced, relapsed or refractory multiple myeloma and renal failure.

## Patients and Methods

### Patients

Twenty consecutive patients (9 male, 11 female, median age = 76.5 years, range 68–85 years) with relapsed (n= 7) or refractory (n = 13) multiple myeloma with disease-related moderate to severe chronic renal failure, defined as creatinine clearance (Cr Cl) ≤ 50 ml/minute (Table 1) received lenalidomide as salvage treatment at 5 Italian Centers. None of the patient had a pre-existent nephropathy such as nephroangiosclerosis, diabetes or AL-amyloidosis. Previous therapy (1–3 lines) included thalidomide in 9 patients (47%), bortezomib in 17 patients (84%) and autologous stem cell transplantation in 2 patients. Thirteen patients were refractory to the last administered therapy, either to bortezomib (10 patients) or to thalidomide (2 patients) or to both bortezomib and thalidomide (one patient). Eleven patients had a more severe impairment of renal function (CrCl < 30ml/min) and four of them were undergoing hemodyalisis.

### Treatment

Lenalidomide was administered on days 1–21 of each 28-days course; dosing was chosen according to the extent of renal impairment, as previously reported._15_ Specifically, two patients with Cr Cl = 50ml/min were treated with full dose (25mg/day), 7 patients with Cr Cl < 30ml/min received 15mg every other day, patients undergoing hemodyalisis were treated at 5mg/day, the remaining patients received 10mg/day. Dexamethasone was used at 20mg/day once a week. All the patients received antithrombotic prophylaxis with either low-molecular weight heparin or aspirin, according to pre-treatment risk of venous thromboembolism, evaluated according to Dimopoulos et al.^16^ Treatment was continued until disease progression or occurrence of grade > = 3 non-hematological toxicity.

### Clinical and Laboratory Evaluation

Physical examination, quality of life assessment, blood cell counts, serum electrolytes, serum levels of immunoglobulins, serum creatinine, creatinine clearance and Bence-Jones proteinuria were evaluated before treatment and every other week thereafter. Toxicity and adverse events occurring during thalidomide therapy were evaluated according to the WHO grading system.

### Assessment of Tumor Response

Response to lenalidomide was assessed after a minimum of 8 weeks; criteria for defining a complete response (CR), a very good partial response (VGPR) a partial response (PR) or a progressive disease (PD) were those reported by the International Myeloma Study Group^17^ with the addition of minor response (MR) category, according to Kyle et al.^18^

### Renal Response

Improvement in renal function, i.e. complete, partial and minor renal response, were evaluated according to recently reported criteria.^19^

## Results

### Response

**Figure 1:**
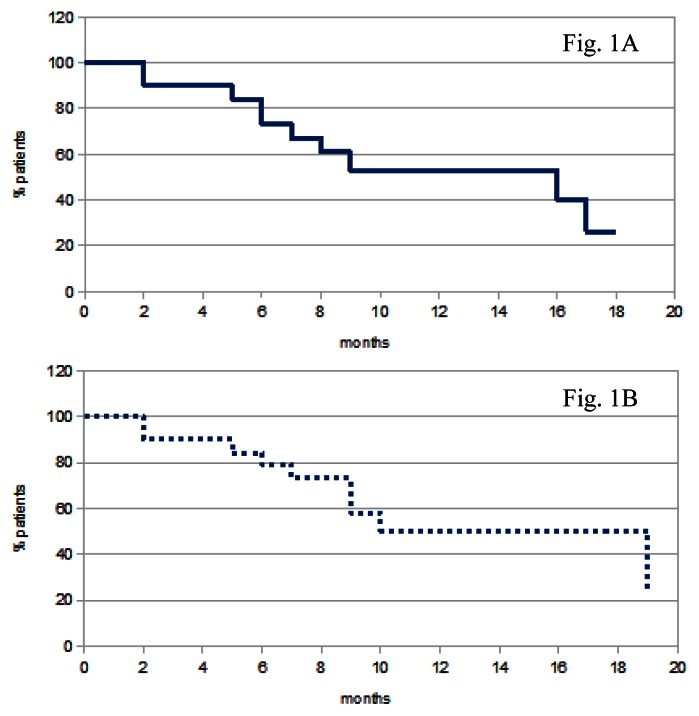
Progression-free (A) and overall survival (B) of treated patients

All the patients but two completed at least 8 weeks of treatment; data were analyzed on an intention – to – treat basis. A ≥ partial response was observed in 8 patients (40%), one of whom obtained a complete response and one a very-good partial response. Four additional patients obtained a minor response, for an overall response rate of 60% (Table 2). Maximal response was achieved after a median of 8 weeks. Median response duration was 16 months (range 2–19+ months) (Figure 1A). Seven patients (35%) were refractory to treatment, even though all of them showed a stable disease lasting an average 7.5 months.

### Renal Response

Recovery of a normal renal function, defined as creatinine clearance ≤ 60ml/min was observed in 4 out of 8 responding patients (20% of the whole population). Three patients showed an increase in CrCl from < 30 to > 30ml/min (partial response). One further responding patient who was dependent on chronic hemodyalysis showed an improvement of renal function and dyalisis was withdrawn (minor response). No improvement of renal function was observed in patients who were refractory to lenalidomide + dexamethasone therapy.

### Toxicity

Toxic effects that were recorded in this series of patients were comparable to those observed in patients with a normal renal function. Neutropenia was the most commonly observed side effect (35% of the patients), although only 3 patients experienced grade ≥3 infections. No patient showed grade ≥3 thrombocytopenia. Dose reduction was necessary in 3 patients while therapy was interrupted after 1 21-day cycle in 2 patients, due to tremors and dizziness (1 patient) and stroke (1 patient), this latter patient received low-molecular heparin prophylaxis. Another patient interrupted the treatment after 5 cycles due to recurrent severe infections

### Patient Status and Survival

After 8 months median follow up, 2/8 responding patients showed disease progression; 1 of them died. Five additional non responding patients have died. Overall survival averages 9 months (Figure 1B). No second primary tumor was observed.

## Discussion

Recently published reports have demonstrated that lenalidomide-based drug combinations are highly effective in in pre-treated advanced MM.^20^ Major concerns were risen regarding the use of the drug in patients with renal failure. Although a direct damage to the kidney has not been demonstrated in MM, worsening of renal function has been described in patients with AL. amyloidosis.^21^ Lenalidomide is excreted by the kidney, so that its clearance decreases in patients with renal failure, with a consequent 6–12 hours increase in plasma half-life and area-under the curve (AUC).^7^ Retrospective evaluation of relapsed/refractory MM patients with some degree of renal impairment treated with full dose Lenalidomide in the context of clinical trials including mainly patients with normal renal function^10^,^11^ confirmed the efficacy of the drug but also the occurrence of hematological toxicity, mainly thrombocytopenia that can potentially lead to more frequent treatment discontinuations. Later reports^12^–^14^ that were mainly focused on patients with renal failure showed that a proper dose reduction can limit hematological toxicity. We further tested this latter hypothesis by analyzing the data obtained in 20 elderly patients with MM and renal insufficiency. Taking into account that the median age of our patients was higher (76.5 years) than that reported by other studies^10^–^11^, ^14^–^15^ the first, and most significant observation, is that the drug appeared to be safe, as side effects and toxicity, both hematological and non hematological, were not different or more severe than those observed in patients with a normal renal function. Even though karyotype evaluation was not carried out in our patients, their prognosis can be classified as poor ( high International staging system score, high percentage of thalidomide and/or bortezomib resistance); in spite of that, 40% of the patients obtained at least a partial response, and this figure is similar to that reported in relapsed-refractory patients with a normal renal function, treated with lenalidomide-dexamethasone combination. 20 Overall, a renal response was achieved in eight patients, all of whom showing some degree of disease response. We can thus assume that renal response is strictly dependent on the attainment of a disease response that is a reduced production of light chains, and not on a direct effect on the inflammatory mechanisms that contribute to the pathogenesis of myeloma kidney as it has been described for bortezomib.^22^ A bortezomib-containing regimen is generally considered the first choice therapeutic approach for patients with renal failure and MM, either newly diagnosed or relapsed/refractory. All the same however, our study and others^10^–^14^ showed that lenalidomide-based drug combination can induce a response even in bortezomib refractory patients, thus indicating that a lenalidomide-based approach should not be overlooked, especially in elderly patients. Oral administration and, above all, the absence of toxic effects that are encountered upon thalidomide or bortezomib therapy, such as lethargy, constipation and peripheral neuropathy, render the drug convenient for long-term use, especially in elderly patients.^23^ Our results, although obtained in a small series of patients, confirm that lenalidomide + dexamethasone can represent a useful therapeutic tool for elderly patients even in case of renal failure, provided an appropriate dose reduction and close monitoring of side effects.

## Figures and Tables

**Table 1:** Patients characteristics

Total Number	20

M/F	9/11

Median age (range)	76.5 yrs (68–85)

IgG isotype (%)	6 (30)

K light chain (%)	12 (60)

Stage III Durie and Salmon	11 (55)

Stage III ISS	15 (75)

Creatinine clearance	

50–30 ml/min (%)	9 (45)

< 30 ml/min (%)	11 (55)

Dialysis (%)	4 (20)

Relapsed	7 (35)

Refractory	13 (65)
Bortezomib refractory	11 (50)
Thalidomide refractory	3 (15)

**Table 2:** Disease response and renal response

Myeloma response	Patient number (%)	Renal response
CR/VGPR	2 (10)	1 complete, 1 minor *
PR	6 (30)	3 complete, 1 partial
MR	4 (20)	2 partial
SD	7 (35)	None
NE	1 (5)	None

* Dyalisis withdrawal
